# Birzeit University Students' Perception of Bottled Water Available in the West Bank Market

**DOI:** 10.1155/2020/5986340

**Published:** 2020-09-27

**Authors:** Buthainah Abdah, Issam A. Al-Khatib, Abdelhaleem I. Khader

**Affiliations:** ^1^Faculty of Graduate Studies, Birzeit University, P.O. Box 14, Birzeit, West Bank, Ramallah, State of Palestine; ^2^Institute of Environmental and Water Studies, Birzeit University, P.O. Box 14, Birzeit, West Bank, Ramallah, State of Palestine; ^3^Civil Engineering Department, An-Najah National University, P.O. Box 7, Nablus, State of Palestine

## Abstract

Water bottling industry has negative environmental impacts due to exploitation and possible pollution of water resources and due to solid waste problems related to the use of plastic bottles. To mitigate these impacts, it is important to study the link between consuming bottled drinking water and the perception of its quality. The objective of the study is to assess the perception of Birzeit University students' of the bottled water marketed in the West Bank and its impact on the humans and the environment. Universities play an important role in providing awareness about environmental issues and sustainability, and university students are thought to be more environmentally conscious about these issues. A quantitative survey was used to analyze the behaviors and perceptions of Birzeit University students. The sample size was 375 students, distributed according to the college, gender, and the academic year at the university. The results show that the factors that affect the perception of the students are mainly the educational year at the university, the income, the family size, and the community type.

## 1. Introduction

The level of perception of the quality of bottled water and its impact on the humans and the environment has an effect on the development of transparency and credibility of the responsible institutions in the aspect of drinking water quality improvement. Media plays an essential role in advertising the bottled water, while education plays a different role in raising the awareness about the quality and negative or positive impacts of bottled water [1].

There are differences in the perception of people in regard to how they assess the quality of bottled water. Some people may assess the quality of bottled water as an end product, and others may assess it in comparison with tap water or other sources of drinking water [2]. The standards which people use to determine the quality of bottled water are useful to judge their perception [3]. For example, if bottled water is more potable and palatable than tap water, then people will tend to think that bottled water is a better choice than tap water [3]. Universities play an important role in providing awareness to the students about the elements of the environment and sustainability principles. University students are thought to be more environmentally conscious than others who tend to adopt a sustainability attitude [4].

Water bottling plants have negative effects on the area in their zones such as streams, rivers, and groundwater. Plastic, in general, and plastic bottles are wastes that represent a major problem in the landfill if it is not recycled [5]. One other impact of the bottled water industry on the environment is the depletion of groundwater and decreasing the flow of streams and lakes due to immense water extraction leading to environmental exhaustion [6].

Few studies have questioned and discussed the link between consuming bottled drinking water and the perception of its quality, while most of the research studies have put their main objectives on the process of production, the regulations, supply and demand, and review and consequences [6]. Many studies showed the differences in gender and the level of education can affect the preference of bottled water over tap water and vice versa based on the variations in the perception of environmental risk [7, 8]. Education, culture, social status, economy, and psychological factors are the causes of risk perception and preventive attitude [9]. Research also shows the relationship between supply and demand of bottled water and environmental awareness. For example, the environmental concern in the U.S. has a role in restricting the consumption of bottled drinking water which can be noted in the drop of sales in the bottled water market in the recent years [6]. Some reasons for choosing bottled water over tap water for drinking purpose are higher quality, better taste, and hazards free [1].

Meanwhile, many conflicts were raised regarding the increasing consumption of bottled drinking water due to its potential negative impacts on the environment either during the filling process or in the wastes produced after consumption [10]. But one cannot neglect the reasons behind the growing consumption of bottled drinking water. For example, an incident of high concentrations of lead found in the municipal (tap) water in Flint, Michigan in the United States, raised safety and health concerns between the public [11]. The main objective of this research was to determine the perception of Birzeit University students of the quality of bottled water and its impact on both humans and environment. It will help to understand the general behavior of the students toward the use of bottled water and to determine the main factors that affect their perception and behavior regarding consuming bottled water.

## 2. Methodology

Data were collected to analyze the perceptions of Birzeit University students of the use of bottled water. The study population was students of Birzeit University (in the academic year 2018/2019).  Table 1 shows the students' distribution at Birzeit University by college and gender for the academic year 2018/2019. The representative sample was measured to be 375 students from a total number of 14,346 students by following the procedure in [12].  Table 2 shows the sample distribution of enrolled students at Birzeit University by college, educational year level, and gender for the academic year 2018/2019.

**Figure 1:**
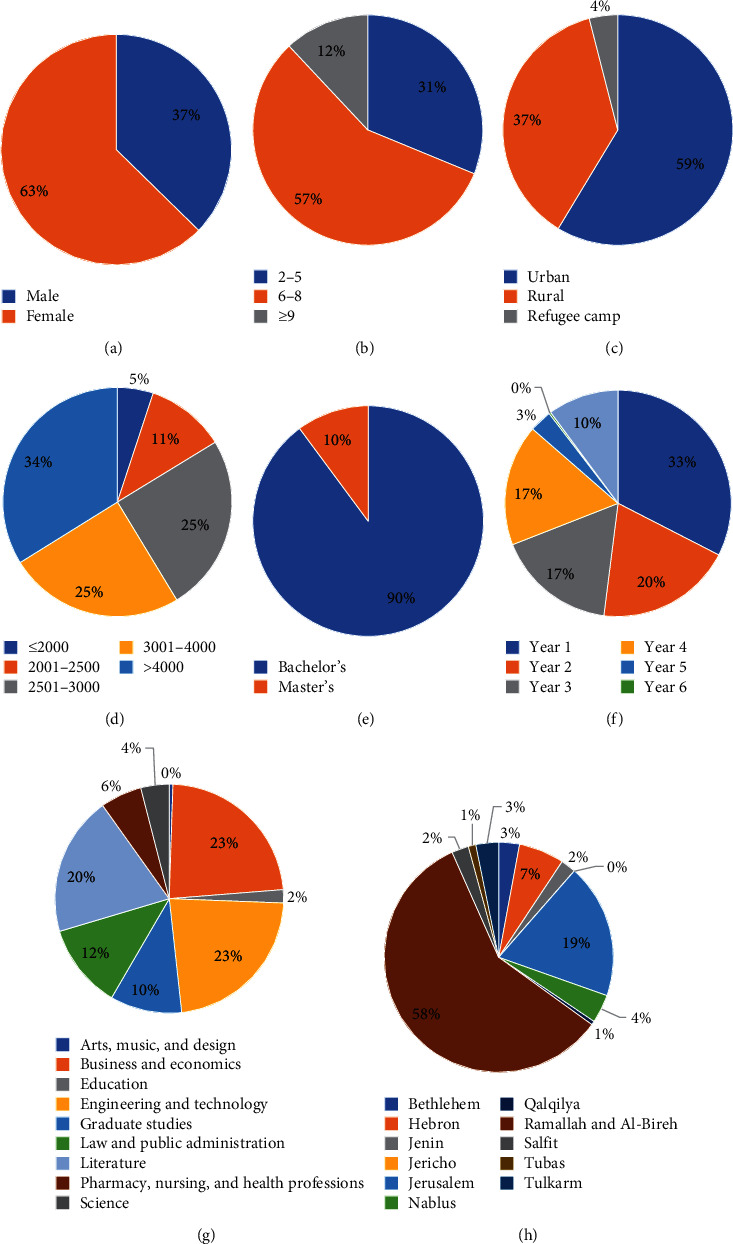
Demographic characteristics of the respondents (independent factors). (a) Gender. (b) Number of family members. (c) Residence type. (d) Average family income (NIS/month). (e) Degree. (f) Year. (g) College. (h) Governorate.

A quantitative survey was used to analyze the behaviors and perceptions of Birzeit University students. Hence, a specifically designed questionnaire was used as a tool for collecting data from a statistically representative sample of students. The questionnaire (Appendix A) was conducted in the Arabic language (Palestinian native language). The questionnaire contained specific parameters for drinking water choices such as health safety, hygiene, convenience and availability, taste, personal and family habits, and environmental concerns. Each of these factors was divided into different subfactors. The sample of the study was distributed according to the college, gender, and academic year at the university.  Figure 1 shows the distribution of the surveyed sample based on gender, number of family members, community type, family income, degree, academic year, college, and governorate. The data were statistically analyzed by Statistical Package for the Social Sciences (SPSS) Statistics version 22.0.

## 3. Results and Discussion

**Figure 2:**
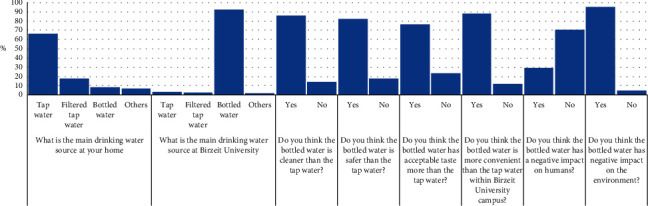
Overall responses of the students to the survey questions (dependent factors).

As indicated in Figure 2, the results show that the main source of drinking water for the families of the students of Birzeit University back at home was the tap water with a percentage of 66.4%, followed by filtered tap water with a percentage of 18.1% and bottled water with a percentage of 8.8%. Meanwhile, 6.7% of the families relied on other drinking water sources such as harvested rainwater and buying water from vendors. Most families depended on tap water which might be an indication for the high quality of tap water (in their opinion) or they cannot afford extra expenses for an external drinking water source. Some people think there is no difference in the quality between tap water and bottled water.

The main drinking water source that Birzeit University students rely on was bottled water with a percentage of 92.0%. The students who consume tap water and filtered tap water were 3.5% and 2.7%, respectively. However, the percentage of the students who used other sources of drinking water at the university was 1.9%, such as bringing tap water or filtered tap water from the drinking water source they use at home.

The outcome of the opinion of Birzeit University students about the bottled water quality in comparison with tap water and the bottled water impact on both human beings and the environment varied between agreement and disagreement. In regard to the quality of bottled drinking water, 85.9% of the students' sample thought that the bottled drinking water is cleaner than tap water and 82.4% of the students agreed that bottled water is safer than tap water. In regard to the taste of bottled water, 76.5% of the students agreed that bottled water has a better taste than tap water. The previous three statements about cleanliness, safety, and taste of bottled drinking water contradicted with the percentage of the main source of drinking water at home, which showed a majority for using tap water as a source of drinking water at home with a percentage of 66.4%. Meanwhile, the three statements go along with the percentage of the main drinking water source within Birzeit University which is 92.0% for bottled water. The reasons for these differences can be explained as follows:

- The drinking water available at home is according to its availability, accessibility, and the financial capacity and options for the families of the students of Birzeit University.
- The students, generally, have the freedom to choose their drinking water source within the Birzeit University campus.
- The ease of access to bottled drinking water (the main choice for the students within the University) is shown in Figure 2. The percentage of the students who agreed that the bottled water is more convenient and easier to reach within Birzeit University was 87.7%.

Regarding the impacts of consuming bottled drinking water, the majority of the students with a percentage of 70.7% thought the bottled drinking water has no effect on humans. Some of the students thought a high concentration of sodium could negatively affect people who suffer from high blood pressure. Other students thought a high concentration of calcium could negatively affect people who suffer from kidney disease. Moreover, few students thought the plastic material of the bottle could seep into the water and negatively affect human health. Regarding the impact of bottled drinking water on the environment, 94.9% of the students agreed that bottled drinking water has an impact on the environment, mostly because of the plastic waste.

A cross-tabulation was applied using SPSS. The purpose of applying cross-tabulation is to determine which of the dependent factors are correlated to the independent factors, within a confidence limit of 95%. If the *P* value is less than 0.05, the factors are not independent of each other, and a statistical relationship between these variables exists.

Regarding the independent factor “gender,” and after performing the ANOVA test, it was found that none of the eight dependent factors shown in Figure 2 were found to be significant to this independent factor, with *P* value >0.05. This indicates that there are no statistically significant differences between male and female students at Birzeit University toward the perception of bottled water available in the West Bank market.

**Figure 3:**
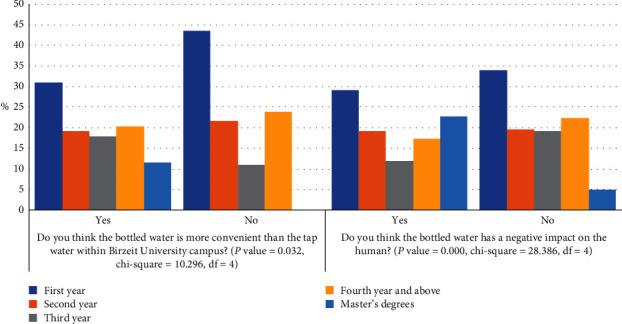
Variation in students' responses based on academic year.

After performing the ANOVA test, only two out of eight dependent factors in Figure 2 were found to be significant to the independent factor “academic year,” with *P* value ˂0.05, as shown in Figure 3. A cross-tabulation test was performed in order to see the effect of the academic year on the dependent factors of perception of the students about the convenience of bottled water within the campus and the impact of bottled drinking water on humans.

Figure 3 shows the variation in students' responses based on the independent factor “academic year.” The relatable dependent factors were found to be “the bottled drinking water is more convenient than the tap water within the Birzeit University campus” and “the bottled drinking water has a negative impact on the human.” The answers varied for both cases for the different academic year levels and the percentage of students who agreed and disagreed was almost equal to each other in some cases.

In respect of the convenience of reaching the bottled water within the campus, it was found that this dependent factor is related to the academic year of the students since *P* value = 0.032 (based on 95% confidence interval). The majority of first-year students thought that the bottled water is less convenient than tap water within the university campus. The same pattern was observed in the second-year students where the students who disagreed were more than the students who agreed. But the students' perspective divided between agreement and disagreement into almost two equal groups. The opinions of the third-year students were reversed since the highest percentage of them agreed that the bottled water is more convenient than tap water within the campus. The opinions of the fourth-year and above students followed the pattern of the second-year students while all of the Master's degrees students agreed that the bottled water is more convenient than the tap water within the university campus. This variation in the opinions for the different academic years comes from many reasons. Many students are not satisfied with a variety of bottled water available in the vending machines or their selling spots because almost all of these vending machines provide one brand of bottled water. So, they prefer bringing tap water from home or drinking from fountain water in the university. Other students trusted the quality of bottled water more than the tap water available at the campus, so they preferred to buy bottled water from its selling spots.

A variation in the opinions has been noticed between agreement and disagreement for “the bottled drinking water has a negative impact on the human” in the different academic years. Most of the students who agreed that the bottled water has a negative effect on the humans were from the first year, followed by the Master's degrees students, second-year students, and fourth year and above students, and third year students in a descending order. However, most of the students who disagreed that the bottled water has a negative effect on the humans were also from the first year, followed by the fourth year and above students, second-year students, third-year students, and the Master's degrees students also in a descending order.

In a study conducted in Suriname to evaluate the consumers buying behavior of bottled drinking water, it was found out that there is no considerable relationship between the behavior of buying bottled drinking water and the demographic variables of education, age, and gender. The consumers also had a positive perception of bottled water quality than tap water since they described it with positive characteristics such as reliable, refreshing, convenient, safer, healthier, available items, socially accepted, and a good substitute to other beverages [13].

In another study, the participants of a students' sample had thought highly of the tap water over the bottled water regarding its quality and the proenvironmental behavior for water consumption. Meanwhile, they confirmed using reusable plastic drinking water bottles to refill them with tap water. In addition, the sales of bottled water were common because of the availability of bottled water selling points at all university facilities, which confirmed the undeniable existence of bottled water [14].

**Figure 4:**
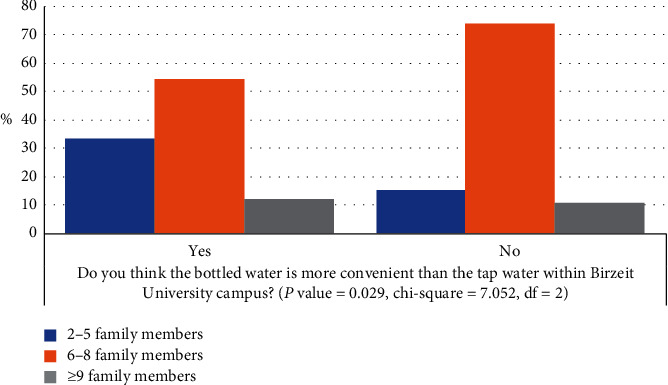
Variation in students' responses based on number of family members.

When the ANOVA test was performed, only one out of eight dependent factors in Figure 2 was found to be significant to the independent factor “number of family members,” with *P* value <0.05, as shown in Figure 4. The cross-tabulation test showed the relationship between the independent factor of “number of family members” and the dependent factor of “bottled drinking water is more convenient than the tap water within the Birzeit University campus,” with *P* value = 0.029 (Figure 4). Most of the students who thought that the bottled water is more convenient than tap water within the university campus have family members between 2 and 5 individuals. However, the highest percentage of the students who have family members more than 9 individuals also agreed. The highest percentage who disagreed was the students who have family members between 6 and 8 individuals.

The results of a study which was carried out in the Philippines showed that the households that came to realize that their different drinking water source at home were to be harmfull preferred to consume bottled water or purified water instead [15]. Factors other than the drinking water safety were found to have a significant effect on buying bottled water, such as the number of individuals in a household, household income, bottled water price, the presence of children younger than 5 years, and the education level of the household heads. However, the income was not a significant factor in deciding to buy or not to buy bottled water [15].

**Figure 5:**
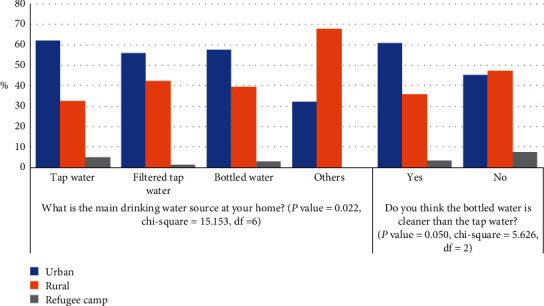
Variation in students' responses based on number of community types.

The ANOVA test showed that only two out of eight dependent factors in Figure 2 were significant to the independent factor “community type,” with *P* value <0.05, as shown in Figure 5. The relation between the independent factor “community type” and the dependent factors “the main drinking water source at home” and “the bottled drinking water is cleaner than the tap water” was analyzed by cross-tabulation, with *P* value equal to 0.022 and 0.05, respectively.

The majority of the students' communities were in the urban areas in the West Bank, followed by the rural areas, and the lowest percentage was of the students who live in the refugee camps. So, the different water sources used at home were chosen by the students mostly from the urban areas. The students who use tap water as the main source of drinking water at home in the urban areas were as twice as the students in the rural areas, while they were the least in the refugee camps. The same pattern was noticed for the homes that use filtered tap water and tap water but with a less gap between the urban and rural areas (around 15%) than that for the houses that use tap water, while the refugee camps have the least percentage. In regard to the homes that use other drinking water sources, the rural areas have the highest percent and then the urban areas come next with almost half of the percentage, while the refugee camps' homes have no other water sources than the tap water, filtered tap water, and bottled water. The highest percentage in the other water sources in the rural areas was mostly because of using wells.

The students who thought the bottled drinking water is cleaner than tap water were mostly from the urban regions in the West Bank while more than half of them were from the rural region and the least were from the refugee camps. However, the students who did not think the bottled water is cleaner than the tap water were almost equal for the urban and rural areas. The high percentage of the students who did not agree on “the bottled water is cleaner than the tap water” in the rural area might be because of comparing their quality with other water sources used at their homes. For example, if the quality of water extracted from wells (which they use as a main source of drinking water) was lower compared with tap water, the idea of “the tap water has a high quality” will be the standard.

In the early 1970s, the consumption of bottled water in the urban areas was much higher than in the other regions in the French cities. That was due to the poor state of the worn lead pipes and the low quality of the urban tap water [16]. Another study between two countries (the United Kingdom and Portugal) was done to compare the effect of the perceptions of drinking water quality and risk on the consumers' behavior. It was found that the people who use bottled water as the main source of drinking water are 53% of the Portuguese respondents and 34% of the United Kingdom respondents [17].

A survey was conducted at an urban clinic, where 208 participants were a convenience sample of caretakers of teenagers and younger generations, regarding their perceptions of the bottled water and tap water qualities, their choices between tap water and bottled water, and their awareness about fluoride [18]. The percentage of participants who depended on bottled water as an only source of drinking water was 38% and the percentage of participants who depended on tap water as an only source of drinking water was 17%, while 42% depended on both bottled drinking water and tap water as a source of drinking water. So, the bottled water was the preferred source of drinking water in the pediatric population at the urban clinic. The driving force over the type of drinking water preferences seemed to be the perceptions of the qualities of the different sources of drinking water [18].

**Figure 6:**
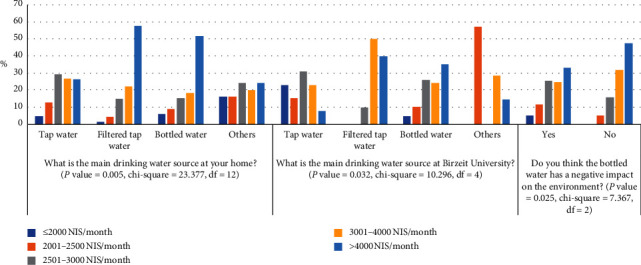
Variation in students' responses based on average family income (NIS/month).

The ANOVA test showed that three out of eight dependent factors in Figure 2 were significant to the independent factor “average family income” with *P* value <0.05, as shown in Figure 6. The relation between the independent factor “average family income” and the dependent factors “the main drinking water source at home,” “the main drinking water source at Birzeit University,” and “the bottled drinking water has a negative impact on the environment” was analyzed by cross-tabulation, with *P* value equal to 0.005, 0.032, and 0.025, respectively.

The highest percentage of students who used tap water at their homes has an average family income in the range of 2501–3000 NIS/month. The highest percentage of students who used filtered tap water and bottled water at their homes has an average family income of more than 4000 NIS/month. The highest percentage of students who used other drinking water sources at their homes has an average family income in the range of 2501–3000 NIS/month and more than 4000 NIS/month.

The highest percentage of students who used tap water in the Birzeit University campus has an average family income in the range of 2501–3000 NIS/month. However, the highest percentage of students who used filtered tap water in the Birzeit University campus has an average family income in the range of 3001–4000 NIS/month. Furthermore, the highest percentage of students who used bottled water in the Birzeit University campus has an average family income more than 4000 NIS/month. The highest percentage of students who used other drinking water sources in the Birzeit University campus has an average family income in the range of 2001–2500 NIS/month.

The highest percentage of the students who agreed that the bottled water has a negative impact on the environment has an average family income of more than 4000 NIS/month, while the least percentage was for the students who have an average family income of less than 2000 NIS/month. However, the highest percentage of the students who disagreed that the bottled water has a negative impact on the environment has an average family income of more than 4000 NIS/month and the least percentage was for the students who have an average family income in the range of 2001–2500 NIS/month. None of the students whose families' income was less than 2000 NIS/month thought that bottled drinking water has an impact on the environment. In general, the factors that affect the students' perception of the bottled water quality and its impact on the humans and the environment are their education level and awareness, the financial status of their families, and their community type.

A study conducted in Parral, Mexico, showed that the willingness of the households to pay for an additional service of drinking water (e.g., bottled water, filtered water, cisterns, etc.), which is reliable and safe, is within the range of 1.8–7.55% above their usual water bill [18]. Considering bottled water as a luxury item, a research study concluded that there is a relation between the income and the behavior of buying bottled water [19]. Independent youngsters and students in the range of 16–25 years old with relatively high-income show tendencies to buy bottled water as a luxurious item they can get any time they want. Even though the people in the range of 16–25 years old usually have low income, yet they are also devoted consumers of bottled drinking water. This category of people is affected by the intense bottled water marketing and the luxury items that are socially accepted [19].

One study conducted in two different universities (University of Vermont and Washington University in St. Louis) to assess the effectiveness of decreasing the plastic waste by banning bottled water showed different results of the ban of bottled water [20]. While the consumption of bottled beverages decreased because of the ban of bottled beverages in some of the studies, the consumption of sugar-sweetened beverages which can cause weight gain has increased in other studies. Two different solutions that were suggested to solve this problem are as follows: partial ban of bottled beverages and adding a plastic bottle tax to the coat [20].

A survey about water quality and safety and preference between bottled water and tap water was conducted in Pennsylvania with a total of 143 participants from the parents of child care centers [20]. The majority of the participants preferred tap water over bottled water for its higher quality and safety in their opinions. They were also concerned over both the impact of bottled water on the environment and the potential pollution resulted from nuclear power plants and the process of natural gas drilling [21].

## 4. Conclusion and Recommendations

The study demonstrated the level of Birzeit University students' perception of bottled water quality and its impact on humans and the environment. A questionnaire was distributed to the students of Birzeit University to assess their perception of bottled water quality and its effect on humans and the environment. The analysis of the data showed that the factors that affect the perception of the students are mainly the educational year at the university, the income, the family size, and the community type. Students with different community types showed a variation in responses with respect to the cleanliness of bottled water in comparison with tap water. So, it could be that the students are aware of the quality of bottled and tap water. The same thing applies to students from different academic years, who had variation of opinions about whether bottled water has a negative effect on humans or not. Also, many students with different family incomes showed variations in opinions on whether the bottled water has a negative impact on the environment. As the education level increases, the awareness about the water quality, in general, will also increase. So, a variety of opinions will be noticed for different students. Also, as the financial status for the family increases, there will be a wide range of options for additional water resources at home and other facilities. The community type may be the main effect on the main water source at home. The supplied water in the urban, rural, and the refugee camps should be of high quality and sampled and tested on a regular basis. And if it was not of high quality or it was not available in sufficient quantities, filters can be applied or another water source can be added if the financial status allowed to.

## Figures and Tables

**Table 1:** Distribution of enrolled students at Birzeit University by college and gender for the academic year 2018/2019.

Enrolled in Bachelor's degree		Gender		Total
		Female	Male	
College	Arts, music, and design	48	29	77
	Business and economics	2,004	1,355	3,359
	Education	264	36	300
	Engineering and technology	1,296	1,928	3,224
	Law and public administration	1,158	584	1,742
	Literature	2,096	696	2,792
	Pharmacy, nursing, and health professions	685	107	792
	Science	469	130	599
	Graduate studies	935	526	1,461
	Total	8,955	5,391	14,346

**Table 2:** Sample distribution of enrolled students at Birzeit University by college, educational year level, and gender for the academic year 2018/2019.

College	Year 1		Year 2		Year 3		Year 4		Year 5		Year 6		Master's degrees		Total
	F	M	F	M	F	M	F	M	F	M	F	M	F	M	
Arts, music, and design	1	1	0	0	0	0	0	0	0	0	0	0	0	0	2
Business and economics	21	14	11	8	10	7	10	6	0	0	0	0	0	0	87
Education	3	0	2	0	1	0	1	0	0	0	0	0	0	0	7
Engineering and technology	11	16	7	10	8	10	5	7	4	7	0	0	0	0	85
Graduate studies	0	0	0	0	0	0	0	0	0	0	0	0	24	14	38
Law and public administration	11	5	6	3	6	3	7	4	0	0	0	0	0	0	45
Literature	21	7	13	4	10	3	12	4	0	0	0	0	0	0	74
Pharmacy, nursing, and health professions	5	1	4	1	3	1	4	1	1	0	1	0	0	0	22
Science	4	1	3	1	2	0	3	1	0	0	0	0	0	0	15
Total	77	45	46	27	40	24	42	23	5	7	1	0	24	14	375

F: female; M: male

## Data Availability

The data used to support the findings of this study are available from the authors upon request.

## Appendix

### A. Questionnaire

The questions in the questionnaire were as follows:

(I) Choose your college
  1. Literature
  2. Arts, music, and design
  3. Business and economics
  4. Education
  5. Engineering and technology
  6. Law and public administration
  7. Pharmacy, nursing, and health professions
  8. Science
(II) Choose your academic year in the university
  1. First year
  2. Second year
  3. Third year
  4. Fourth year and above
  5. Graduate studies (Master and Ph.D.)
(III) Choose your gender
  1. Male
  2. Female
(IV) State the number of your family members __________
(V) State which city you are from _________ and choose one of city/village/refugee camp
(VI) Choose your family's monthly income
  1. Less than or equal to 2000 NIS
  2. 2001–2500 NIS
  3. 2501–3000 NIS
  4. 3001–4000 NIS
  5. More than 4000 NIS
(VII) Choose the main drinking water source at your home
  1. Tap water
  2. Filtered tap water
  3. Bottled water
  4. Others
(VIII) Choose the main drinking water source at Birzeit University
  1. Tap water
  2. Filtered tap water
  3. Bottled water
  4. Others
(IX) Do you think bottled water is cleaner than tap water?
  1. Yes
  2. No
(X) Do you think bottled water is safer than tap water?
  1. Yes
  2. No
(XI) Do you think bottled water has acceptable taste more than tap water?
  1. Yes
  2. No
(XII) Do you think bottled water is more convenient than tap water within the Birzeit University campus?
  1. Yes
  2. No
(XIII) Do you think the bottled water has a negative impact on the human?
  1. Yes
  2. No
(XIV) Do you think the bottled water has a negative impact on the environment?
  1. Yes
  2. No
